# Leveraging Single-Cell and Spatial Transcriptomics in Gut Nutrition and Mucosal Immunology

**DOI:** 10.1016/j.tjnut.2026.101668

**Published:** 2026-06-11

**Authors:** Jiaxing Yang, Nan Gao

**Affiliations:** 1 Department of Biological Sciences, Rutgers University, Newark, NJ, United States; 2 Department of Pharmacology, Physiology and Neurosciences; Center for Immunity and Inflammation, Rutgers Health-New Jersey Medical School, Newark, NJ, United States

**Keywords:** single-cell RNA sequencing, spatial transcriptomics, inflammatory bowel disease, infection, cell–cell communication

## Abstract

In this editorial, we describe how single-cell transcriptomics is advancing the field of intestinal nutrition biology, as highlighted in recent studies. These studies have uncovered potential communication between epithelial and immune cells, generating innovative hypotheses regarding nutrient metabolism in the context of mucosal immunology. We also discuss how emerging spatial transcriptomics and in vivo labeling models may propel conventional technologies forward, in terms of uncovering physical cell–cell interactions and spatial cell–cell communication that can be modulated by nutrient intervention.

The intestine represents one of the most complex immunological tissue compartments in the human body, serving as an interface between the host and dietary antigens, commensal microbes, and various pathogens. Acute enteric infections by invasive bacteria, parasitic helminths, or viruses trigger localized and systemic defense responses, transforming the gut into an immunologically active environment. Infected epithelial cells secrete antimicrobial peptides and proinflammatory cytokines to combat pathogens and activate innate and adaptive immune cells. These intercellular communications and interactions also occur under chronic inflammatory conditions such as during the pathogenesis of the inflammatory bowel diseases (IBD), where the compromised epithelial barrier and a hyperactivated immune system drive progressive tissue damage.

To understand these complex and dynamic interactions across diverse cell populations in response to infection and inflammation, high-throughput approaches are required. Single-cell RNA sequencing (scRNA-seq) has revolutionized our ability to understand gene expression dynamics across various cell types and to uncover functions of poorly characterized cell populations. Haber et al. [1] provided one of the earliest comprehensive single-cell surveys of the mouse small intestinal epithelium, characterizing gene expression profiles in ileal epithelial cells and revealing tuft cell subpopulations. Building upon this, Elmentaite et al. [2] generated a large integrated atlas of the human intestinal tract. They comprehensively mapped the diversity of intestinal epithelial cells, discovered the BEST4^+^ cells, and delineated the development of the enteric nervous system and the formation of secondary lymphoid structures in the gut. In addition to these baseline characterizations, subsequent scRNA-seq studies have profiled epithelial and immune cell populations including Paneth cells, CD8^+^ T cells, macrophages, and neutrophils from healthy individuals and patients with IBD or bacterial infections [[3], [4], [5], [6], [7], [8]]. These studies offered insights into the mechanisms of inflammation and how the gut responds to pathogenic challenges.

Harnessing the power of these technologic advances in studying dietary nutrition, a recent study by Tang et al. [9], published in *The Journal of Nutrition*, offered high-resolution insights into the genetic link between nutrient metabolism and immune regulation. The authors established the first integrated single-cell atlas of the mouse ileum in the context of nutrient absorption and mucosal immunity. This study comprehensively investigated nutrition absorption and metabolism across different cell types in the ileum, providing insights into the interplay between gut epithelial and immune cells. The authors revealed distinct subpopulations characterized by specialized programs for carotenoid metabolism, vitamin A absorption, and vitamin B12 absorption. These findings illustrate the heterogeneity of enterocytes, suggesting that distinct enterocyte subsets may play specialized roles in processing various nutrient cues. Intriguingly, the authors identified plasmacytoid dendritic cells (pDCs) as a potential central mediator of epithelial–immune cell interactions and further uncovered a novel co-expression pattern between β-carotene oxygenase 2 (Bco2) and interleukin-18 (IL-18) in enterocytes and a subset of goblet cells. These genes are closely localized on the same chromosome, in both human and mouse. Building upon this discovery, Tang et al. [9] further validated that Bco2 deficiency reduced IL-18 expression in goblet cells. Goblet cells are classically known for secreting mucin and for their active transport of dietary antigens across the intestinal barrier to antigen-presenting cells, primarily dendritic cells (DCs) [10]. How goblet cells recruit DCs and the exact factors regulating these processes remain poorly understood. Findings by Tang et al. [9] may indicate IL-18 as a chemoattractant secreted by goblet to recruit pDCs [11]. Thus, the work may have uncovered a previously unrecognized mechanism underlying goblet cell-associated antigen passage, offering a hypothesis that nutrition may critically regulate the interplay between epithelial and immune cells.

This work highlights that intestinal cell–cell communication (CCC) is an intricate, rapidly growing field of investigation that bridges the dietary biology and mucosal immunology. Because no single intestinal cell functions alone, high-throughput CCC studies may focus on long-range signaling, e.g., through secreted ligands and cell-surface receptors, or direct physical interactions among neighboring cells. The nature of these cell–cell interactions dictates distinct biological processes, including growth factor-stimulated wound healing, stem cell proliferation and differentiation, and antigen presentation. In conventional scRNA-seq analyses, CCC inference largely relies on ligand–receptor expression patterns across cell types, utilizing tools with curated databases such as CellChat and CellPhoneDB [12]. However, due to the lack of spatial information, these approaches cannot assess the physical feasibility of interactions based on the spatial proximity of cells. As a result, predicted interactions may not occur in vivo due to spatial constraints. Given the presence of intraepithelial lymphocytes and extensive immune–epithelial contact events, e.g., antigen presentation or transcytosis in epithelial cells, physiological cell–cell contacts must be investigated to fully elucidate mechanisms of immune regulation in health and disease.

Spatial transcriptomics has emerged to offer a platform for characterizing the tissue niches or microenvironments containing various cell types. It enables researchers to assess the spatial organization of cells and infer their physical proximity to each other. Applying this technology to IBD pathology has yielded transformative insights exemplified by Kong et al. [13]. Their team revealed a localized fibrosis-associated cellular network that drives tissue scarring and stricture formation in Crohn’s disease [13]. Likewise, Battat et al. [14] mapped out distinct inflammatory signatures across different anatomical regions in patients with Crohn’s disease. Xu et al. [15] applied spatial transcriptomics to identify potential murine homologs of human BEST4^+^ cells and uncovered significant changes in immune cell abundance by comparing conventional and germ-free mice to evaluate the impact of the microbiome.

For further investigations of CCC under healthy and diseased conditions, next-generation computational tools for spatial transcriptomics analysis, such as COMMOT (COMMunication analysis by Optimal Transport) and NiCo, enable precise modeling of ligand diffusion and reveal microenvironment-specific communication between epithelial and immune cells [16,17]. However, there remains a lack of studies using these tools to address how immune–epithelial interactions change during inflammation or how the altered interactions influence inflammatory disease progression. Additionally, these bioinformatic predictions of CCC require experimental validation. For example, the LIPSTIC (labeling immune partnerships by SorTagging intercellular contacts) mouse models that directly label cells in physical contact with a target cell type of interest could help advance this area of research [18].

In summary, it is essential to integrate spatially informed models with in vivo experimental validations to deconvolute the complex crosstalk between the epithelium, the immune system, and the microbiota during infection and inflammation. The gap between transcriptomic predictions and functional interactions needs to be closed by integrated multimodal approaches. Insight gained through systems biology and bench research will inform targeted therapeutic strategies for gastrointestinal diseases.

## Author contributions

The authors’ responsibilities were as follows – JY, NG: prepared the first draft; and both authors: read and approved the final manuscript.

## Declaration of generative AI and AI-assisted technologies in the writing process

During the preparation of this work, the authors used Gemini for language editing assistance. After using this tool/service, the authors reviewed and edited the content as needed and take full responsibility for the content of the publication.

## Funding

The authors were supported by CCFA SRA 1456013; NIH R01DK132885; NIH R01DK139790.

## Conflict of interest

The authors reported no conflict of interest.
